# Space Robot Sensor Noise Amelioration Using Trajectory Shaping

**DOI:** 10.3390/s24020666

**Published:** 2024-01-20

**Authors:** Emily Kuck, Timothy Sands

**Affiliations:** 1 Sibley School of Mechanical and Aerospace Engineering, Cornell University, Ithaca, NY 14853, USA; epk37@cornell.edu; 2 Department of Mechanical Engineering (SCPD), Stanford University, Stanford, CA 94305, USA

**Keywords:** structural dynamics, flexible robotics, bandpass filter, notch filter, structural filtering, trajectory generation, whiplash compensation

## Abstract

Robots in space are necessarily extremely light and lack structural stiffness resulting in natural frequencies of resonance so low as to reside inside the attitude controller’s bandwidth. A variety of input trajectories can be used to drive a controller’s attempt to ameliorate the control-structural interactions where feedback is provided by low-quality, noisy sensors. Traditionally, step functions are used as the ideal input trajectory. However, step functions are not ideal in many applications, as they are discontinuous. Alternative input trajectories are explored in this manuscript and applied to an example system that includes a flexible appendage attached to a rigid main body. The main body is controlled by a reaction wheel. The equations of motion of the flexible appendage, rigid body, and reaction wheel are derived. A benchmark feedback controller is developed to account for the rigid body modes. Additional filters are added to compensate for the system’s flexible modes. Sinusoidal trajectories are autonomously generated to feed the controller. Benchmark feedforward whiplash compensation is additionally implemented for comparison. The method without random errors with the smallest error is the sinusoidal trajectory method, which showed a 97.39% improvement when compared to the baseline response when step trajectories were commanded, while the sinusoidal method was inferior to traditional step trajectories when sensor noise and random errors were present.

## 1. Introduction

**Figure 1:**
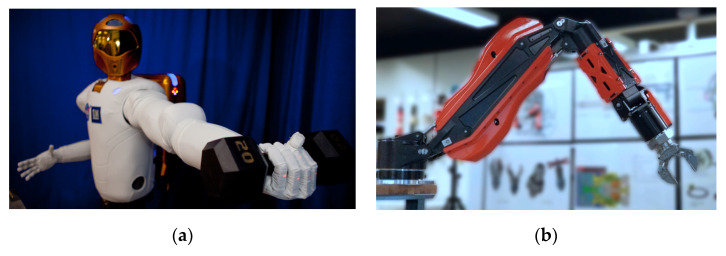
(**a**) NASA’s Robonaut 2, built at Johnson Space Center, became the first android astronaut to go to space in 2011. Now, about two dozen former NASA engineers, many of whom helped build the robot astronaut, have turned their skills to creating underwater robots at Nauticus Robotics [1]; (**b**) Nauticus is also commercializing the robotic arm technology–known as Olympic Arm–that it developed while designing and building Aquanaut. Image credits (both): Nauticus Robotics Inc., Webster, IA, USA [1] used in compliance with image use policy [2], “NASA content (images, videos, audio, etc.) are generally not copyrighted and may be used for educational or informational purposes without needing explicit permissions”.

The solutions and methods developed in this manuscript are applicable to a wide range of dynamics problems. A robotic arm is chosen here as an example of a highly flexible system, with implications across the industry. Robots are deployed in space for a variety of purposes. Figure 1a shows one such robot, NASA’s Robonaut 2. In addition to space applications, robotic arms can be deployed underwater, as evidenced by Figure 1b. Whether in space or underwater, the commanded trajectory can influence the tracking error of the robotic arm.

Minimizing tracking error can allow for more precise movements and support the execution of detailed motion. In-orbit servicing is an example of the need for precise control over a robotic arm’s movement. By identifying the most appropriate trajectory to command, the error in angular movement can be minimized.

Towards in–orbit servicing, the importance of spacecraft-mounted robotics missions is highlighted in reference [3] which stipulates:

“…environmental, economic, and strategic considerations support the claim that the future of a space infrastructure will depend on the ability to perform on-orbit servicing, encompassing a broad array of in-space operations, such as inspection, berthing, refueling, repair, assembly, and so on.”

According to a 2010 study by the U.S. National Air and Space Administration (NASA), [4] a key to enabling robotic servicing missions in space leading to advantageous future strategic impact, cost effectiveness, and environmental sustainability. Cost effectiveness is manifest in the ability to relatively cheaply replace spacecraft components rather than launch a replacement spacecraft. Reference [5] indicates since 1957 roughly 5400 space missions have been flown, while nearly twenty-thousand space objects are tracked by the north American air defense command (NORAD), where over two thousand are rocket upper stages spent of fuel and over ten thousand additional items are classified as debris. Current proposals [6,7] indicate intentions for very large future constellations together comprising another twenty thousand objects in orbit. Such a large number of craft in orbit constitute a potentially lucrative business model for system repair [8] and refueling on-orbit. Discovery of the very origins of life and human long-term habitability are postulated to be aided by space robotics in [9].

This manuscript investigates the importance of the trajectory shaping fed to the control method used to rotate the space robot. The flexible spacecraft system examined in this manuscript is indicative of a larger dynamics problem. The solutions and methods explored are applicable to that larger set of dynamic problems. The requisite equations of motion are derived and feedback controllers and second-order structural filters are applied, following the methodology developed in [10]. Initially, sinusoidal trajectory generation is used to drive the controllers. Whiplash compensation is additionally investigated as a solution, per [11]. Three methods of trajectory shaping are applied to the flexible spacecraft robotic system and compared critically: step shaped, sinusoidally shaped, and whiplash shaped. The state errors, rate errors, and control efforts are compared for each of the three methods of input control to determine which is most appropriate for the flexible spacecraft system application.

Elder techniques for controlling highly flexible systems relied foremost on feedback necessitating construction of feedback linearizing control laws [12]. The linear-quadratic regulator approach, robust control approaches minimizing the H∞ and H2 norms, and a disparate approach based in analytical dynamics, introduced as the Udwadia–Kalaba approach were compared in reference [13]. Very recently, integration of fuel slosh with centralized sensors and actuators, without the usage of collocated devices for vibration management. into techniques to control the motion of flexible appendages was offered by [14] reiterating the relevance of classical proportional, derivative (PD) control with nonadaptive bandpass filters, where the novel proposition includes integration of wave-based control with the filtered PD control scheme. Vibration suppression was illustrated by establishing a dynamic grasping area to describe the contact procedure of the capture device grasping target in reference [15]. Control of rotation-floating space robots with flexible appendages specifically for on-orbit servicing was proposed in [16]. Techniques were suggested using a composite two-time-scale control system [17]. Open-loop methods were strictly used for analysis while closed-loop was utilized for control in reference [18]. Another alternative is adaptive feedback control [19].

The following list highlights the current state of the art developing deterministic artificial intelligence:

1. In 2019, reference [11] revealed an optimal control revealed by pseudospectral optimization software where the solution validation was provided using six theoretical necessary conditions of optimization: (1) Hamiltonian minimization condition; (2) adjoint equations; (3) terminal transversality condition; (4) Hamiltonian final value condition; (5) Hamiltonian evolution equation; and lastly (6) Bellman’s principle. The results are novel and unique in that they initially command full control in the opposite direction from the desired end state, while no such results are seen using classical control methods including classical methods augmented with structural filters typically employed for controlling highly flexible multi-body systems.
2. Later in 2022, an interesting study of the use of feedback and structural filtering to maximize system stability was offered [10], leading to the recommendation to use single-sinusoidal trajectory shaping to maximize stability.

The following brief list articulates the novel growth from the current state of the art methods.

1. Rather than propose options for maximizing stability [10], this study seeks to offer advice to minimize trajectory tracking errors.
2. Rather than focus on feedforward [11] versus feedback [10], this study investigates commanded trajectory tracking options.

This manuscript advises the readership on methods to shape the commanded trajectory to be tracked, where sinusoidal, whiplash, and step trajectories are critically compared using tracking errors (both angle and angular rate) and control effort as figures of merit.

## 2. Materials and Methods

**Figure 2:**
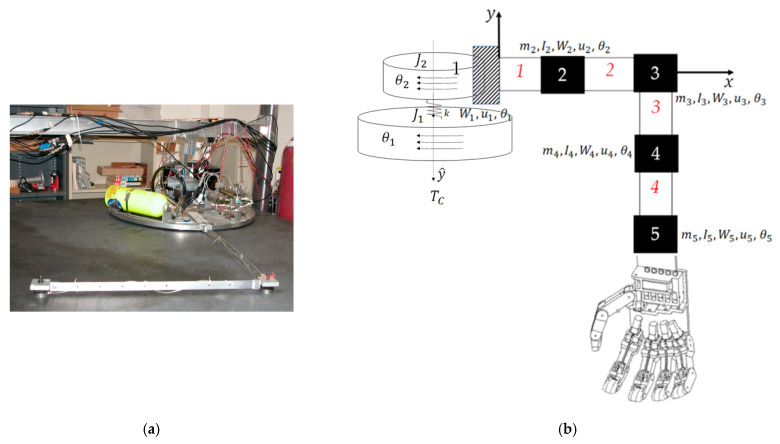
Flexible spacecraft system. (**a**) Atop a (planar) air bearing table, a free-floating space robot is autonomously controlled. [20] Imagery and photographs of the Department of Defense are in the public domain, unless otherwise noted [21]; (**b**) schematic diagram of the free-floating flexible space system depicted in subfigure (**a**). This schematic is identical to that used in references [10,11], where this manuscript comprises the latest iteration of continuing research. No special permission is required to reuse all, or part of the article published by MDPI, including figures and tables [22].

In this manuscript, the flexible spacecraft system shown in Figure 2 is analyzed. The system consists of a rigid main body R, reaction wheel W, and a flexible appendage F. The flexible appendage F is split into beam elements 1 through 7, and node points 1 through 8. Table 1 lists parameters of the flexible spacecraft system and their descriptions. The methods applied in this section can be used for any dynamic, flexible system and is not limited to spacecraft application. The flexible spacecraft system in Figure 2 is explored in this manuscript as an example.

### 2.1. Equations of Motion

The equations of motion of the flexible spacecraft system are derived using the Lagrange method. Lagrangian Mechanics requires kinetic and potential energies. See Table 1 for parameter definitions.

The kinetic energy of the entire flexible spacecraft system was found by summing the kinetic energies of the rigid main body, flexible appendage, and reaction wheel. The kinetic energies are written in terms of the moments of inertia and the modal coordinates, as seen in Equation (1). $D_{i}$ are the rigid-elastic coupling terms and are defined in Equation (2). The modal coordinates are expressed in terms of *q* and *F*, which are defined in Equations (4) and (5) for a given beam *i*.

(1) $$T=\frac{1}{2}I_{zz}{\dot{\theta }}^{2}+\frac{1}{2}I_{w}{{\dot{\theta }}_{w}}^{2}+I_{w}{\dot{\theta }\dot{\theta }}_{w}+\frac{1}{2}\sum_{i=1}^{n}{\dot{q}}_{i}+\dot{\theta }\sum_{i=1}^{n}{D_{i}\dot{q}}_{i}$$

The potential energy is shown in Equation (3), where $\omega_{i}$ are the natural frequencies of the flexible spacecraft system for mode *i*.

(2) $$D_{i}=\int [x_{F}{\phi_{i}}^{y}-y_{F}{\phi_{i}}^{x}]dm$$

(3) $$V=\frac{1}{2}\sum_{i=1}^{n}{\omega_{i}}^{2}{q_{i}}^{2}$$

(4) $$\left\{q\right\}={\left\{\begin{array}{ccc} W_{1} & \theta_{1} & \begin{array}{cc} W_{2} & \theta_{2} \end{array} \end{array}\right\}}^{T}$$

(5) $$\left\{F\right\}={\left\{\begin{array}{ccc} Q_{1} & M_{1} & \begin{array}{cc} Q_{2} & M_{2} \end{array} \end{array}\right\}}^{T}$$

The Lagrange equation, shown in Equation (6) is applied to the Lagrangian *L*, where *L* = *T* − *V*. The Lagrange method results in the equations of motion (EOM) of the flexible spacecraft system, which are shown in Equation (7).

(6) $$\frac{-d}{dt}\left(\frac{\delta L}{\delta {\dot{u}}_{i}}\right)-\left(\frac{\delta L}{\delta u_{i}}\right)=Q_{i}$$

(7) $$EOM:\left\{\begin{array}{c} I_{zz}\ddot{\theta }+I_{w}{\ddot{\theta }}_{w}+\sum_{i=1}^{n}D_{i}{\dot{q}}_{i}=T_{D}\\ I_{w}({\ddot{\theta }}_{w}+\ddot{\theta })=T\\ {\ddot{q}}_{i}+{\omega_{i}}^{2}D_{i}\ddot{\theta }=0 \end{array}\right.$$

### 2.2. Natural Frequencies

After reformulating Equation (7) into canonical form elaborated in Equations (19)–(23) in reference [10], the natural frequencies of the flexible spacecraft system were derived using the finite element method and by solving the eigenvalue problem using the stiffness and mass matrices. It was assumed that all displacements are normal, and the system is constrained in Nastran. The stiffness (*k*) and mass (*m*) matrices are constructed for each beam element. The individual stiffness and mass matrices are shown for a given beam element i in Equations (8) and (9).

(8) $$\left[k^{i}\right]=\left[\begin{array}{cccc} 12 & 6L & -12 & 6L\\ 6L & 4L^{2} & -6L & 2L^{2}\\ -12 & -6L & 12 & -6L\\ 6L & 2L^{2} & -6L & 4L^{2} \end{array}\right]$$

(9) $$\left[m^{i}\right]=\left[\begin{array}{cccc} 156 & 22L & 54 & -13L\\ 22L & 4L^{2} & 13L & -3L^{2}\\ 54 & 13L & 156 & -22L\\ -13L & -3L^{2} & -22L & 4L^{2} \end{array}\right]$$

The individual stiffness and mass matrices are added by superposition to form the total stiffness and mass matrices respectively, which are presented in the Appendix A. The solution to the eigenvalue problem, presented in Equation (10), provides the natural frequencies and mode shapes of the flexible spacecraft system. Recall the expressions for the rigid–elastic coupling using modal coordinates: in accordance with Equation (2), *φ*’s are mode shapes from finite element analysis using the eigenvalues of *K*/*M* (stiffness/mass). The system stiffness matrix is included in Equation (8) and mass matrix in Equation (9) and result in the natural frequencies and mode shapes for the flexible system. The resulting natural frequencies are listed in Table 2, and their corresponding mode shapes can be found in the Appendix A.

### 2.3. PID Controller

Facilitating the study of input trajectory shaping methods, some control must be benchmarked. While whiplash control from the prequel in reference [11] was selected as the single feedforward control benchmark (to be discussed shortly in Section 2.6), a PID controller with structural filters was chosen as the single benchmark feedback control and was designed to control the motion of the reaction wheel using the procedures in the prequel (Equations (29)–(38) in reference [10]). The PID controller was designed to meet the following specification requirements: 15% overshoot and control bandwidth of 4 rad/s. It is assumed that the natural frequency of the closed loop response is equal to the control bandwidth. The rise time, damping ratio, settling time, and period are calculated according to Equations (10), (11), (12), and (13), respectively.

(10) $$t_{r}=\frac{1.8}{\omega_{n}}=\frac{1.8}{4}=0.45\;s$$

(11) $$\zeta =\frac{-ln(0.15)}{\sqrt{\pi^{2}+{ln}^{2}(0.15)}}=0.517$$

(12) $$t_{s}=\frac{4.6}{{\zeta \omega }_{n}}=\frac{4.6}{0.517*4}=2.22\;s$$

(13) $$T≅\frac{10}{{\zeta \omega }_{n}}=\frac{10}{0.517*4}=4.84$$

The proportional, integral, and derivative gain values are calculated according to Equations (14)–(16).

(14) $$K_{P}=I_{w}\left({\omega_{n}}^{2}+\frac{2{\zeta \omega }_{n}}{T}\right)=1.537$$

(15) $$K_{I}=\frac{I_{w}{\omega_{n}}^{2}}{T}=0.301$$

(16) $$K_{D}=I_{w}\left(\frac{1}{T}+2{\zeta \omega }_{n}\right)=0.396$$

The PID controller and the flexible spacecraft system were modeled in MATLAB^®^, and the simulation results are presented in Section 3.

### 2.4. Second-Order Structural Filters

After the addition of the PID controller, it was determined that additional filtering was needed to compensate for the system’s flexible modes. Additional filtering was added in the form of second-order structural filters. Classical second-order structural filters were designed to compensate for the flexible modes, following the convention defined in Equation (17), where $\omega_{z}$ and $\omega_{p}$ are the frequencies of the zeros and poles respectively and $\zeta_{z}$ and $\zeta_{p}$ are the damping ratios of the zeros and poles. A tutorial elaboration of classical filter design is available in reference [23].

(17) $$\frac{Output(s)}{Input(s)}=\frac{\frac{s^{2}}{{\omega_{z}}^{2}}+\frac{2\zeta_{z}}{{\omega_{z}}^{2}}s+1}{\frac{s^{2}}{{\omega_{p}}^{2}}+\frac{2\zeta_{p}}{{\omega_{p}}^{2}}s+1}$$

Equation (17) was used to generate bandpass and notch filters to compensate for the valley and peaks for each of the flexible mode cantilever responses.

### 2.5. Sinusoidal Trajectory Generation

The feedback controller was commanded by an autonomously generated sinusoidal trajectory to achieve the desired behavior. A piecewise function was created to support the desired quiescent and maneuver times. The generated sinusoid is structured according to Equation (18). Table 3 lists the proximal variable definitions.

(18) $$z=(A-A_{0})[1+\sin\left(\omega t+\emptyset \right)]$$

The frequency, ω, is directly and inversely proportional to the desired time of the maneuver. By increasing the frequency, a faster maneuver time can be achieved. During the quiescent periods, a constant signal will be applied. The final piecewise trajectory is formed by summing the constant signals during the quiescent periods with the sinusoidal function as it traverses one valley to the next peak. This sinusoidal trajectory generation technique was added to the MATLAB^®^ SIMULINK^®^ project and the results are detailed in Section 3.

### 2.6. Whiplash Compensation

Whiplash compensation was proposed as a solution to the flexible spacecraft system control problem in [2]. To prevent overshoot, [2] proposed a driving function that creates motion in the opposite direction as the desired final position.

The whiplash compensation trajectory generation scheme was implemented in SIMULINK^®^ and follows the format of Equation (19).

(19) $$z=(A-A_{0})[\sin\left(\omega t+\emptyset \right)]$$

**Figure 3:**
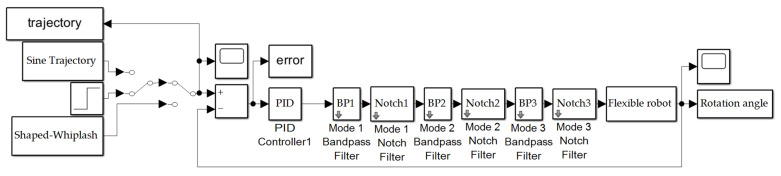
Flexible spacecraft system simulation created in SIMULINK^®^. Subsystems are displayed in Appendix B.

The flexible spacecraft system was simulated using MATLAB^®^’s SIMULINK^®^. A variable-step size was used along with MATLAB^®^’s automatic solver selection. Figure 3 shows the topography of the flexible spacecraft system SIMULINK^®^ model. The input trajectory options are shown on the left part of the figure and include a sine trajectory, a square wave, and the shaped-whiplash trajectory. The PID controller and second order filters are applied, and have been described in Section 2.3 and Section 2.4, respectively. The final rotation angle is examined, and its performance assessed Section 3.

The state and rate sensor errors are introduced in the SIMULINK^®^ model in order to mimic realistic performance. The state and rate sensor errors are defined by a normally distributed random number with 0 mean and 0.01 variance. The sample time used is 0.01 for the state, rate, and inertia error values. The seed value is generated as a uniformly distributed random number.

The simulation was performed 1000 times for Monte Carlo analysis. Each simulation included the state and rate sensor noises. The Monte Carlo analysis was performed for each of the three trajectory generation schemes: (1) step function, (2) sinusoid trajectory, and (3) whiplash compensation.

## 3. Results

**Figure 4:**
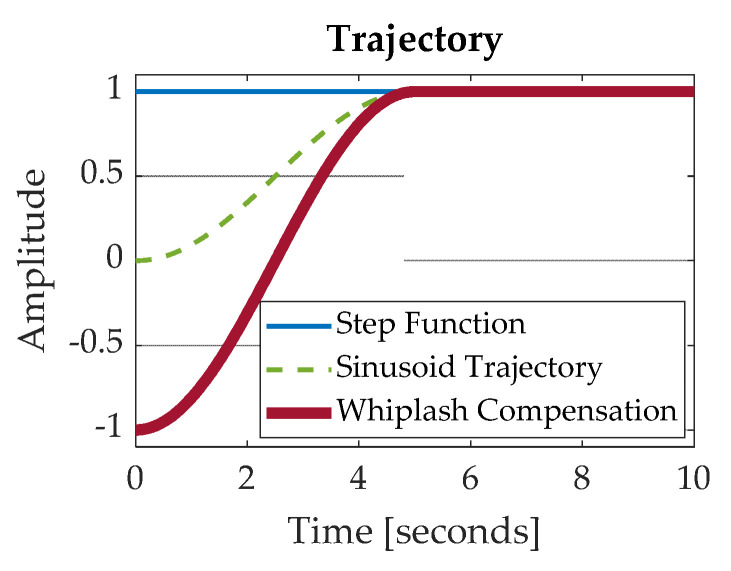
Generated Trajectories.

The various control methods were applied in MATLAB^®^/SIMULINK^®^ in three different trials, where the following inputs were used to drive the system controller: (1) step response, (2) sinusoidal trajectory, and (3) whiplash compensation. Figure 4 depicts each trajectory as a function of time. The system is expected to complete its maneuver by *t* = 5 s. As seen in Figure 4, the step function has an instantaneous change in value because the step occurs at *t* = 0 s. The sinusoid and whiplash trajectories, on the other hand, are not instantaneous and have a duration. The sinusoid trajectory starts at an amplitude of 0, while the whiplash trajectory starts at an amplitude of −1. The whiplash method generates motion in the opposite direction as the final desired state to prevent overshoot.

**Figure 5:**
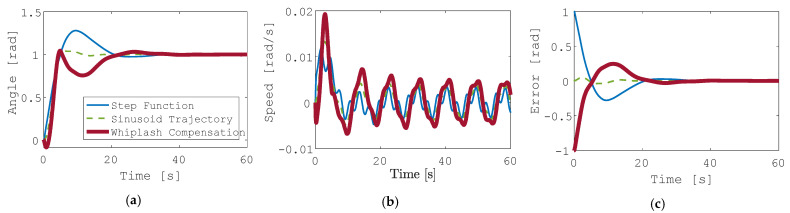
Generated trajectories (**a**) reaction wheel angle θ; (**b**) reaction wheel speed $\dot{\theta }$; (**c**) reaction wheel rotation angle error.

The flexible spacecraft system is driven by each of the three trajectories in Figure 4. The resulting angle of the reaction wheel is plotted in Figure 5b for all three cases. Similarly, Figure 5c shows the reaction wheel speed error for each controller method. In each figure, the response to a step function input is shown in blue, the response to the generated sinusoid is shown in a dashed green line, and the response to the whiplash compensator is shown in a bolded red line. Figure 5 depicts these results without state and rate sensor noise included.

The mean error value and standard deviation of the error value was calculated and is tabulated in Table 4. Surprisingly, the sinusoid trajectory response shows less error than either the step response or the whiplash response. Monte Carlo simulations were performed for each of the three input trajectory generation schemes. There were 1000 Monte Carlo trials executed in each simulation. There are two sensor noises included in the model: rotation angle state noise and rotation angle rate noise. Three combinations of noise were included in the Monte Carlo trials: (1) state noise only, (2) rate noise only, and (3) both state and rate noise.

**Figure 6:**
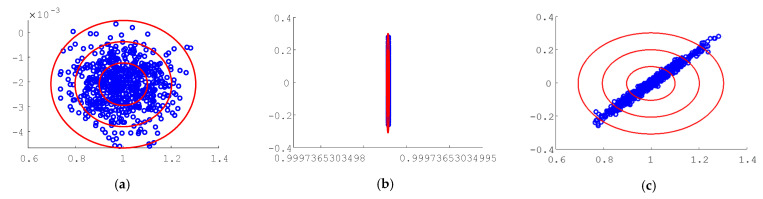
Monte Carlo analysis of random perturbations for the step function input trajectory, final rotation angle value is on the abscissa and final rotation angle rate value is on the ordinant, each blue dot represents one Monte Carlo trial (**a**) space robot rotation angle in the presence of angle sensor noise; (**b**) space robot rotation rate in the presence of angle rate sensor noise; (**c**) space robot rotation rate in the presence of both angle sensor noise and angle rate sensor noise.

Figure 6 depicts the shotgun plot analysis for each of the noise combinations for the step function input trajectory. Each dot represents one Monte Carlo trial. The one-, two-, and three-sigma ellipses are depicted in red. Table 5 details the mean and standard deviations for each of the methods and combinations of sensor noise.

## 4. Discussion

The mean and standard deviation values are compared to the baseline step response error values and tabulated as percentage differences in Table 6. The sinusoid trajectory method shows the most improvement, with an error value 97.39% closer to the desired trajectory than the step response. The whiplash compensation method, on the other hand, proves to be less accurate than the step response, showing a 42.16% increase in mean error.

Driving the flexible spacecraft system with a sinusoidal trajectory is the solution with minimal error when sensor noise is not included. The sinusoidal trajectory generation scheme creates a near step response trajectory, without the discontinuities associated with a step response. The whiplash response is similarly continuous however, it shows more errors than the baseline step response. These results confirm the whiplash solution as a suboptimal result, as was first proposed in [2].

When normally distributed random sensor noise is added to the state and rate sensors, the results align more closely to real world applications. The Monte Carlo simulations performed are summarized in Table 7.

## 5. Conclusions

The whiplash trajectory generation scheme shows a 133.3% improvement when only rate sensor noise is included. The step function method has the least mean error in rotation angle when only the state sensor noise is enabled, as well as when both rate and state sensor noises are enabled.

Without noise included, the sinusoid trajectory shows the most improved performance. However, in the presence of state and rate sensor noises, the step function has the smallest mean error values for the rotation angle. When noise is included, the step function has a 13.64% improvement in mean error when compared to the sinusoid trajectory.

Future research should be conducted to investigate more optimal, continuous trajectory schemes. This could also include investigating the most appropriate solver in MATLAB^®^. Depending on the solver and step size chosen in MATLAB^®^’s SIMULINK^®^, the resulting trajectories could produce different results. Investigating which solver and step size is the most appropriate for this application would render additional confidence in the results. Additionally, more trajectory generation schemes could be evaluated and compared to the sinusoid and whiplash methods. Evaluating additional methods could lead to a more optimal solution with less error.

## Figures and Tables

**Table 1:** Flexible Spacecraft System Parameters.

Variable	Definition	Variable	Definition	Variable	Definition
T	Kinetic energy	T	Natural frequency of i-th mode	V	Potential energy
$I_{zz}$	Moment of inertia of rigid body	$\phi_{i}$	Modal coordinates	W	Displacement
$I_{w}$	Moment of inertia of reaction wheel	$x_{F}$	Final position, x	Q	Sheer forces
θ	Angle of flexible appendage	$y_{F}$	Final position, y	M	Moments
$\theta_{w}$	Angle of reaction wheel	n	Number of independent modes	L	Lagrangian
$q_{i}$	Modal coordinates	$D_{i}$	Elastic decoupling coefficients	$T_{D}$	Disturbance torque

**Table 2:** Natural Frequencies, $\omega_{n}$ for the Flexible Spacecraft System, in rad/s.

1809.46	596.81	43.72	10.22
1415.52	478.77	30.89	2.07
1042.16	419.02	15.77	0.69
774.31	54.87		

**Table 3:** Table of proximal variables and nomenclature ^1^.

Variable/Acronym	Definition	Variable/Acronym	Definition
z	Sinusoidal trajectory	ω	Frequency
A	Desired magnitude	t	Time
$A_{0}$	Initial magnitude	∅	Phase offset

^1^ Such tables are offered throughout the manuscript to aid readability.

**Table 4:** Reaction wheel error values. “e−4” notation indicates “${\times 10}^{–4}$”.

Method	Rotation Angle θ Error Mean	Rotation Angle θ Error Standard Deviation
Step	0.0102	0.2051
Sinusoid	−2.66e−4	0.0141
Whiplash	−0.0145	0.2097

**Table 5:** Monte Carlo analysis of random perturbations. “e−4” notation indicates “${\times 10}^{–4}$”.

Method	Rotation Angle θ Error Mean	Rotation Angle θ Error Standard Deviation	Rotation Angle θ Error Mean	Rotation Angle θ Error Standard Deviation	Rotation Angle θ Error Mean	Rotation Angle θ Error Standard Deviation
	With Rotation Angle Sensor Noise		With Rotation Angle Rate Sensor Noise		With Rotation Angle Sensor and Rate Sensor Noise	
Step	−0.0005	−0.0021	−0.0003	−0.0044	−0.0022	−0.0039
Sinusoid	−0.0016	−1.9424e−4	−2.7023e3	16.7673	−0.0025	−0.0019
Whiplash	−0.0007	0.0017	0.0001	−8.8541e−4	−0.0059	−0.0054

**Table 6:** Percent errors for reaction wheel angle.

Method	Rotation Angle θ Error Mean	Rotation Angle θ Error Standard Deviation
Step	---	---
Sinusoid	−97.39%	0.0141
Whiplash	42.16%	0.2097

**Table 7:** Monte Carlo analysis of random perturbations (percent performance improvement).

Method	Rotation Angle θ Error Mean	Rotation Angle θ Error Standard Deviation	Rotation Angle θ Error Mean	Rotation Angle θ Error Standard Deviation	Rotation Angle θ Error Mean	Rotation Angle, θ Error Standard Deviation
	With Rotation Angle Sensor Noise		With Rotation Angle Rate Sensor Noise		With Rotation Angle Sensor and Rate Sensor Noise	
Step	---	---	---	---	---	---
Sinusoid	220.0%	−90.75%	$900\times 10^{6}$%	−381$\times 10^{3}$%	13.64%	−51.28%
Whiplash	40.00%	−181.0%	−133.3%	−79.88%	168.2%	38.46%

**Figure A1:**
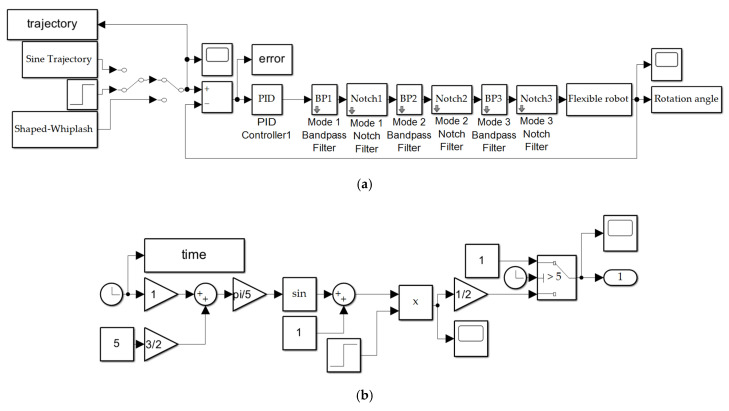

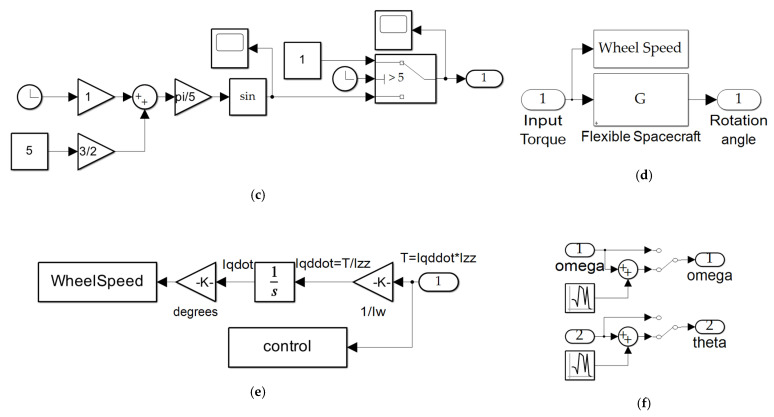
SIMULINK^®^ models used to produce the results in this manuscript. (**a**) actuator reaction wheel simulation subsystem; (**b**) noisy sensor subsystem, (**c**) sinusoidal trajectory generation, (**d**) subsystem for flexible space robot, (**e**) conversion of wheel torque to display wheel speed, (**f**) sensor noise added to both angular state and rate measurements.

## Data Availability

To access the data, please contact the corresponding author.

## Appendix A

**Table A1:** Stiffness Matrix, [K]. “e−4” notation indicates “${\times 10}^{–4}$”.

	$W_{2}$	$\theta_{2}$	$W_{3}$	$\theta_{3}$	$W_{4}$	$\theta_{4}$	$W_{5}$	$\theta_{5}$	$u_{6}$	$\theta_{6}$	$u_{7}$	$\theta_{7}$	$u_{8}$	$\theta_{8}$
$W_{2}$	958.818	0	−479.41	59.926	0	0	0	0	0	0	0	0	0	0
$\theta_{2}$	0	19.975	−59.926	4.9938	0	0	0	0	0	0	0	0	0	0
$W_{3}$	−479.41	−59.926	958.82	0	−479.41	59.926	0	0	0	0	0	0	0	0
$\theta_{3}$	59.926	4.9938	0	19.975	−59.926	4.9938	0	0	0	0	0	0	0	0
$W_{4}$	0	0	−479.41	−59.926	958.82	0	−479.41	59.926	0	0	0	0	0	0
$\theta_{4}$	0	0	59.9260	4.9938	0	19.975	−59.926	4.9938	0	0	0	0	0	0
$W_{5}$	0	0	0	0	−479.41	−59.926	479.41	−59.926	0	0	0	0	0	0
$\theta_{5}$	0	0	0	0	59.926	4.9938	−59.926	19.975	−59.926	4.9938	0	0	0	0
$u_{6}$	0	0	0	0	0	0	0	−59.926	958.82	0	−479.41	59.926	0	0
$\theta_{6}$	0	0	0	0	0	0	0	4.9938	0	19.975	−59.926	4.9938	0	0
$u_{7}$	0	0	0	0	0	0	0	0	−479.41	−59.926	958.82	0	479.41	59.926
$\theta_{7}$	0	0	0	0	0	0	0	0	59.926	4.9938	0	3.39e−5	−59.926	4.9938
$u_{8}$	0	0	0	0	0	0	0	0	0	0	−479.41	−59.926	479.41	−59.926
$\theta_{8}$	0	0	0	0	0	0	0	0	0	0	59.926	4.9938	−59.926	9.9877

**Table A2:** Mass Matrix, [M]. “e−4” notation indicates “${\times 10}^{-4}$.

	$W_{2}$	$\theta_{2}$	$W_{3}$	$\theta_{3}$	$W_{4}$	$\theta_{4}$	$W_{5}$	$\theta_{5}$	$u_{6}$	$\theta_{6}$	$u_{7}$	$\theta_{7}$	$u_{8}$	$\theta_{8}$
$W_{2}$	0.4760	0	0.0037	−2.2e−4	0	0	0	0	0	0	0	0	0	0
$\theta_{2}$	0	3.39e−5	2.2e−4	−1.27e−5	0	0	0	0	0	0	0	0	0	0
$W_{3}$	0.0037	2.2e−4	0.4760	0	0.0037	−2.2e−4	0	0	0	0	0	0	0	0
$\theta_{3}$	−2.2e−4	−1.27e−5	0	3.39e−5	2.2e−4	−1.27e−5	0	0	0	0	0	0	0	0
$W_{4}$	0	0	0.0037	2.2e−4	0.4760	0	0.0037	−2.2e−4	0	0	0	0	0	0
$\theta_{4}$	0	0	−2.2e−4	−1.27e−5	0	3.39e−5	2.2e−4	−1.27e−5	0	0	0	0	0	0
$W_{5}$	0	0	0	0	0.0037	2.2e−4	2.63	−3.73e−4	0	0	0	0	0	0
$\theta_{5}$	0	0	0	0	−2.2e−4	−1.27e−5	−3.73e−4	3.39e−5	2.2e−4	−1.27e−5	0	0	0	0
$u_{6}$	0	0	0	0	0	0	0	2.2e−4	0.4760	0	0.0037	−2.2e−4	0	0
$\theta_{6}$	0	0	0	0	0	0	0	−1.27e−5	0	3.39e−5	2.2e−4	−1.27e−5	0	0
$u_{7}$	0	0	0	0	0	0	0	0	0.0037	2.2e−4	0.4760	0	0.0037	−2.2e−4
$\theta_{7}$	0	0	0	0	0	0	0	0	−2.2e−4	−1.27e−5	0	3.39e−5	2.2e−4	−1.27e−5
$u_{8}$	0	0	0	0	0	0	0	0	0	0	0.0037	2.2e−4	0.4660	−3.73e−4
$\theta_{8}$	0	0	0	0	0	0	0	0	0	0	−2.2e−4	−1.27e−5	−3.73e−4	1.69e−5

**Table A3:** Mode Shapes for each Natural Frequency. “e−4” notation indicates “${\times 10}^{–4}$”.

1.07e−4	−2.55e−4	−2.32e−4	−8.22e−6	3.08e−4	−5e−4	2.67e−4	0.15	0.0383	0.104	0.0443	−0.0692	−0.024	0.0181
0.11	−0.308	−0.448	−0.499	−0.451	0.317	−0.115	−0.455	−0.0136	0.239	0.222	−0.405	−0.167	0.14
9.94e−5	−7.34e−5	3.19e−4	6.74e−4	4.71e−4	1.36e−4	−2.44e−4	−0.157	−0.0215	0.0204	0.0667	−0.143	−0.0712	0.067
0.216	−0.486	−0.396	−0.0028	0.381	−0.488	0.221	−0.0943	−0.204	−0.706	−0.0867	−0.0912	−0.186	0.246
8.64e−5	0.00014	5.08e−4	−7.13e−7	−7.17e−4	2.86e−4	2.09e−4	0.13	−0.0125	−0.108	0.0125	−0.0995	−0.106	0.139
0.311	−0.45	0.106	0.499	0.119	0.448	−0.314	0.581	0.212	0.0369	−0.257	0.406	−0.0683	0.32
2.43e−5	1.39e−05	4.69e−05	−6.04e−5	−5.41e−5	−9.16e−5	7.14e−05	−0.0099	0.00299	0.0113	−0.0105	0.0248	−0.0954	0.225
0.39	−0.22	0.486	0.00468	−0.488	−0.207	0.394	−0.429	−0.343	0.45	0.188	0.475	0.169	0.365
4.84e−5	3.19e−4	−4.01e−4	−1.45e−5	0.00059	6.16e−4	1.07e−4	0.0536	−0.0918	0.0135	0.0898	0.0754	0.0736	0.0946
0.449	0.106	0.314	−0.499	0.306	−0.123	−0.452	0.0815	0.229	−0.242	0.309	0.0891	0.4	0.389
2.55e−5	2.06e−4	−4.67e−4	6.73e−4	−6.69e−4	−3.84e−4	−4.63e−5	−0.0294	0.0753	−0.0446	0.0881	0.041	0.191	0.194
0.487	0.39	−0.213	−0.00747	0.223	0.392	0.483	−0.247	0.339	−0.021	−0.366	−0.339	0.518	0.401
1.33e−4	1.61e−4	−2.12e−4	2.94e−4	−4.02e−4	−5.22e−4	−6.19e−4	0.00905	−0.0261	0.0192	−0.0699	−0.0732	0.325	0.295
0.503	0.505	−0.501	0.503	−0.506	−0.498	−0.497	0.358	−0.782	0.394	−0.765	−0.515	0.55	0.405
